# Evaluation of Bronchoalveolar Lavage Fluid from Patients in an Outbreak of E-cigarette, or Vaping, Product Use–Associated Lung Injury — 10 States, August–October 2019

**DOI:** 10.15585/mmwr.mm6845e2

**Published:** 2019-11-15

**Authors:** Benjamin C. Blount, Mateusz P. Karwowski, Maria Morel-Espinosa, Jon Rees, Connie Sosnoff, Elizabeth Cowan, Michael Gardner, Lanqing Wang, Liza Valentin-Blasini, Lalith Silva, Víctor R. De Jesús, Zsuzsanna Kuklenyik, Cliff Watson, Tiffany Seyler, Baoyun Xia, David Chambers, Peter Briss, Brian A. King, Lisa Delaney, Christopher M. Jones, Grant T. Baldwin, John R. Barr, Jerry Thomas, James L. Pirkle

**Affiliations:** ^1^Division of Laboratory Sciences, National Center for Environmental Health, CDC; ^2^Office of the Director, National Center for Chronic Disease Prevention and Health Promotion, CDC; ^3^Office on Smoking and Health, National Center for Chronic Disease Prevention and Health Promotion, CDC; ^4^Office of the Director, National Institute for Occupational Safety and Health, CDC; ^5^Office of Strategy and Innovation, National Center for Injury Prevention and Control, CDC; ^6^Division of Overdose Prevention, National Center for Injury Prevention and Control, CDC.

CDC, the Food and Drug Administration (FDA), state and local health departments, and multiple public health and clinical partners are investigating a national outbreak of e-cigarette, or vaping, product use–associated lung injury (EVALI). Based on data collected as of October 15, 2019, 86% of 867 EVALI patients reported using tetrahydrocannabinol (THC)-containing products in the 3 months preceding symptom onset (*1*). Analyses of THC-containing product samples by FDA and state public health laboratories have identified potentially harmful constituents in these products, such as vitamin E acetate, medium chain triglyceride oil (MCT oil), and other lipids (*2*,*3*) (personal communication, D.T. Heitkemper, FDA Forensic Chemistry Center, November 2019). Vitamin E acetate, in particular, might be used as an additive in the production of e-cigarette, or vaping, products; it also can be used as a thickening agent in THC products (*4*). Inhalation of vitamin E acetate might impair lung function (*5*–*7*).

Bronchoscopy and bronchoalveolar lavage^†^ (BAL) can be part of the clinical and diagnostic workup of EVALI patients. The decision to perform this procedure is made by the clinical team on a case-by-case basis (*8*). During August–October 2019, BAL fluid specimens were collected by clinical teams caring for hospitalized EVALI patients. Public health laboratories and health departments from 10 states (California, Connecticut, Hawaii, Illinois, Maryland, Michigan, Minnesota, Texas, Utah, and Wisconsin) coordinated the submission of residual BAL fluid specimens from 29 patients to CDC.

To better characterize exposure among EVALI patients, CDC developed and validated isotope dilution mass spectrometry methods to analyze specific toxicants of concern and active compounds in case-associated BAL fluid.^§^ These CDC analytic methods can identify vitamin E acetate, MCT oil (medium chain triglycerides), plant oils (long chain triglycerides), petroleum distillates (including mineral oil), diluent terpenes, cannabinoids, and nicotine in BAL fluid. The quality of case-associated BAL specimens was assessed by measuring dipalmitoylphosphatidylcholine (DPPC), the principal phospholipid in naturally-occurring lung surfactant: the presence of acceptable levels of DPPC confirms that the lavage procedure recovered adequate pulmonary epithelial fluid. When specimen volume was insufficient to perform all planned analyses, analysis of vitamin E acetate and cannabinoids was prioritized. Among the 27 BAL fluid specimens with sufficient volume for testing, all had measurable levels of DPPC. Overall, 21 (72%) patients with available specimens were male, and their median age was 23 years (range = 16–67 years), which is consistent with the sex and age patterns of EVALI patients reported to CDC to date (*1*). Two of the patients died.

Vitamin E acetate was detected in all 29 patient BAL samples. Among 23 patients for whom self-reported THC use information was available, 20 reported using THC-containing products. THC or its metabolites were detected in 23 of 28 patient BAL samples, including in those of three patients who said they did not use THC products. Nicotine metabolites were detected in 16 of 26 patient BAL specimens. Results for plant oils, MCT oil, petroleum distillates, and diluent terpenes were all below analyte-specific levels of detection (typically in the low ng/mL range).

This is the first reported identification of a potential toxicant of concern (vitamin E acetate) in biologic specimens obtained from EVALI patients. These findings provide direct evidence of vitamin E acetate at the primary site of injury among EVALI patients and are consistent with FDA product testing and media reports of state public health laboratory testing documenting vitamin E acetate in product samples used by EVALI patients (*2*,*3*) (Personal communication, D.T. Heitkemper, FDA Forensic Chemistry Center, November 2019). Other diluents and additives of concern (e.g., plant oils, MCT oil, petroleum distillates, and diluent terpenes) were notably not detected in BAL fluid specimens from EVALI patients.

Although vitamin E acetate was detected in all specimens in this analysis of a convenience sample of 29 EVALI case-associated BAL specimens, additional studies are needed, including comparison with BAL fluid specimens from healthy volunteers and animal studies using controlled exposures to establish whether a causal link exists between this exposure and EVALI. Based on these data from 29 patients, it appears that vitamin E acetate is associated with EVALI; however, it is possible that more than one compound or ingredient could be a cause of lung injury, and evidence is not yet sufficient to rule out contribution of other toxicants to EVALI.

These findings reinforce CDC’s recommendation that persons should not use e-cigarette, or vaping, products containing THC, especially those obtained from informal sources such as friends or family, or those from the illicit market, where product ingredients are unknown or can be highly variable (*9*). Until the relationship of vitamin E acetate and lung health is better characterized, it is important that vitamin E acetate not be added to e-cigarette, or vaping, products. CDC will continue to update guidance, as appropriate, as new data become available from this outbreak investigation.

## Footnotes

† Bronchoalveolar lavage, performed in the evaluation of lung disease, involves instillation of sterile saline into a subsegment of the lung, followed by suction and collection of the fluid for analysis.

§ CDC has not yet published these validated isotope dilution mass spectrometry methods.
