# *Erratum:* Vol. 68, No. 45

**DOI:** 10.15585/mmwr.mm6904a6

**Published:** 2020-01-31

**Authors:** Benjamin C. Blount, Mateusz P. Karwowski, Maria Morel-Espinosa, Jon Rees, Connie Sosnoff, Elizabeth Cowan, Michael Gardner, Lanqing Wang, Liza Valentin-Blasini, Lalith Silva, Víctor R. De Jesús, Zsuzsanna Kuklenyik, Cliff Watson, Tiffany Seyler, Baoyun Xia, David Chambers, Peter Briss, Brian A. King, Lisa Delaney, Christopher M. Jones, Grant T. Baldwin, John R. Barr, Jerry Thomas, James L. Pirkle, Lung Injury Response Team, Lung Injury Response Team, Christina Brosius, Kevin Caron, Carolina Fernandez, Cory Holder, Rudy Johnson, Cody Newman, Chris Reese, Denise Tevis, Dana Meaney Delman, Kara Polen, Anita Patel, Bobby N. Brewer, Macarena Garcia, Ian Williams, Kevin Chatham-Stephens, Jennifer Freed, Donald Hayes, Matt Ritchey

**Affiliations:** ^1^Division of Laboratory Sciences, National Center for Environmental Health, CDC; ^2^Office of the Director, National Center for Chronic Disease Prevention and Health Promotion, CDC; ^3^Office on Smoking and Health, National Center for Chronic Disease Prevention and Health Promotion, CDC; ^4^Office of the Director, National Institute for Occupational Safety and Health, CDC; ^5^Office of Strategy and Innovation, National Center for Injury Prevention and Control, CDC; ^6^Division of Overdose Prevention, National Center for Injury Prevention and Control, CDC.; Division of Laboratory Sciences; National Center for Environmental Health,; CDC; Division of Birth Defects; and; Infant; Disorders; National Center on Birth Defects; and; Developmental; Disabilities; CDC; National Center for Immunization; and; Respiratory; Diseases; CDC; Battelle Memorial Institute, Columbus, Ohio; Center for Surveillance, Epidemiology; and; Laboratory Services,; CDC; National Center for Emerging; and; Zoonotic Infectious Diseases; CDC; National Center on Birth Defects; and Developmental Disabilities; CDC; Division of Community Health Investigations; Agency for Toxic Substances; and Disease Registry; National Center for Chronic Disease Prevention and Health Promotion; CDC.

In the report “Evaluation of Bronchoalveolar Lavage Fluid from Patients in an Outbreak of E-cigarette, or Vaping, Product Use–Associated Lung Injury — 10 States, August–October 2019” names of the members of the Lung Injury Response Team and persons in Acknowledgments were omitted. The names are included below.

